# Superior Mesenteric Vein Occlusion Causing Severe Gastrointestinal Haemorrhage in Two Paediatric Cases

**DOI:** 10.1155/2012/964845

**Published:** 2012-11-06

**Authors:** Anna L. Fox, Matthew Jones, Andrew Healey, Marcus K. H. Auth

**Affiliations:** ^1^Department of Paediatric Gastroenterology, Alder Hey Children's NHS Foundation Trust, Eaton Road, Liverpool L12 2AP, UK; ^2^Department of Paediatric Radiology, Alder Hey Children's NHS Foundation Trust, Eaton Road, Liverpool L12 2AP, UK; ^3^Department of Paediatric General Surgery, Alder Hey Children's NHS Foundation Trust, Eaton Road, Liverpool L12 2AP, UK

## Abstract

Reports about superior mesenteric vein thrombosis in childhood are very rare and have not been associated with gastrointestinal bleeding. We describe two cases of severe bleeding from the upper and lower gastrointestinal tract in children who had undergone complex abdominal surgery at considerable time before. The first child had a tracheoesophageal fistula, corrected by division, gastrostomy insertion, and repair of duodenal rupture. The child presented with severe bleeding from the gastrostomy site and was diagnosed with a thrombosis of the proximal superior mesenteric vein. The second child had a gastroschisis and duodenal atresia, and required duodenoplasty, gastrostomy insertion, hemicolectomy, and adhesiolysis. The child presented with intermittent severe lower gastrointestinal bleeding, resulting from collateral vessels at location of the surgical connections. He was diagnosed with a thrombosis of the superior mesenteric vein. In both children, the extensive previous surgery and anastomosis were considered the cause of the mesenteric thrombosis. CT angiography confirmed the diagnosis in both cases, in addition to characteristic findings on endoscopy. Paediatricians should suspect this condition in children with severe gastrointestinal bleeding, particularly in children with previous, complex abdominal surgery.

## 1. Introduction

Superior mesenteric vein (SMV) thrombosis is an uncommon venous thromboembolic event. It first appears in the medical literature at the end of the 19th Century, identified as a cause of intestinal gangrene by Elliot, who described his surgical experiences in cases of mesenteric venous and arterial occlusion [1].

SMV thrombosis has been described in numerous hypercoagulable states, including pregnancy and oral contraceptive pill use, nephrotic syndrome, and hereditary thrombophilias, malignancy, and in the postoperative period. Infective causes, particularly intra-abdominal sepsis, have also been identified as a cause for mesenteric venous thrombosis. Idiopathic, or primary, SMV thrombosis is less common but is described in a small number of case reports [2].

Abdominal pain, vomiting, diarrhea, and intestinal bleeding are the common presenting features in these cases [3]. Raised mesenteric venous pressure proximal to the thrombotic occlusion gives rise to varices and intestinal oedema. At its most severe, mesenteric vein thrombosis can cause acute intestinal ischaemia and necrosis requiring extensive bowel resection and is a surgical emergency. Mortality for such procedures is high and the resulting morbidity from short bowel syndrome, prolonged periods of intensive care treatment, and parenteral nutrition and its associated complications is significant. 

Morbidity associated with mesenteric thrombosis is exacerbated by the frequent delay in diagnosis. Diagnosis has been greatly improved by the development of more sensitive and advanced imaging techniques. CT angiography is widely considered to be the most useful diagnostic test. Other investigations to identify any underlying pathology such as inherited thrombophilia are also important in the ongoing management of these patients. 

SMV thrombosis is described in a large number of case reports involving adult patients, including some recent papers describing up to fifty patients, but the literature relating to SMV thrombosis in children is limited to a small number of single case reports. To our knowledge, for the first time in paediatrics, we present two cases of SMV thrombosis as the cause of severe gastrointestinal bleeding at a surgical site. 

## 2. Case Presentations

*Case 1*. This female preterm infant was born at nearly 28 weeks gestational age. Intrauterine growth restriction had been diagnosed in the antenatal period and she was born with very low birth weight. She required immediate cardiorespiratory support and a tracheoesophageal fistula (TOF) was diagnosed on day 1 of life. On day 2 of life she underwent surgical repair of the TOF and further surgery on day 4 for division of TOF, gastrostomy insertion, and repair of duodenal rupture. She developed neonatal hyperbilirubinaemia which required phototherapy. She also had a patent ductus arteriosus (PDA) and patent foramen ovale (PFO). Her PDA was ligated at 10 weeks of age or 38 weeks corrected gestational age. 

As a result of her prematurity she developed complex neonatal problems including intraventricular haemorrhage, retinopathy of prematurity, chronic lung disease of prematurity, and metabolic bone disease which led to spontaneous fractures. She had endocrine abnormalities including hypothyroidism and hypoadrenalism which required hormone supplementation. Blood samples were sent for chromosomal analysis but no chromosomal or genetic syndrome was diagnosed. 

Her progress was hindered by intercurrent sepsis and haemodynamic instability. She spent no more than a few days at a time without invasive mechanical ventilation. She achieved full enteral feeding following the placement of a jejunostomy tube via her gastrostomy. There were issues with leakage and recurrent bleeding from her gastrostomy site which at times became difficult to control and led to the need for multiple blood transfusions and pressure dressings. On several occasions, surgical haemostasis with diathermy had to be applied to stop the bleeding. Blood tests during this time showed low platelets and routine coagulation tests including INR, prothrombin time, and APTT were largely normal. 

The recurrence of bleeding from her gastrostomy site and the appearance of prominent veins on the patient's abdomen raised the clinical suspicion of portal hypertension. An ultrasound scan performed when the patient was 26 weeks old (corrected gestation age—term + 14 weeks) showed no liver abnormalities and normal blood flow in the portal and hepatic veins. MRI of her abdomen 2 weeks later failed to find a cause for her bleeding, showing patent portal veins and normal appearances of intra-abdominal organs. 

**Figure 1:**
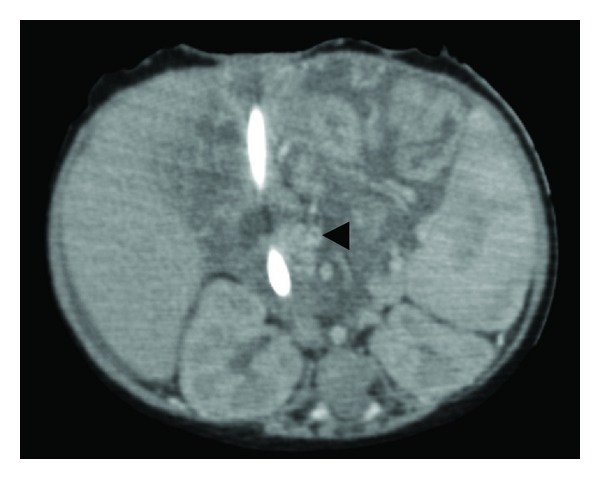
Clot in SMV seen as filling defect with halo of contrast at arrow head.

As her bleeding continued intermittently a CT angiogram was performed. This identified a filling defect—shown in Figure 1—in the proximal superior mesenteric vein. The occlusion was judged to be thrombotic in nature.

**Figure 2:**
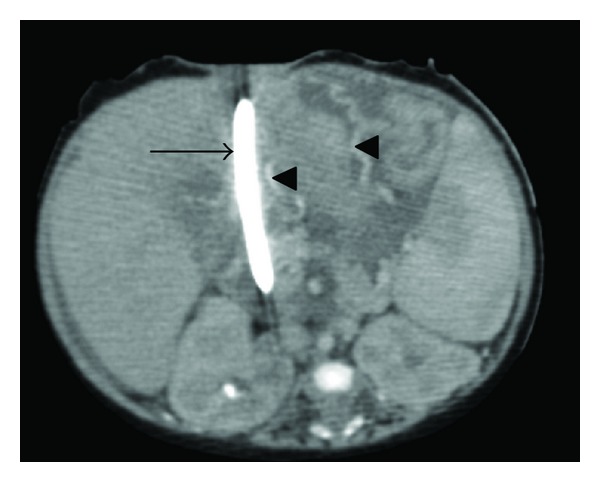
Post IV contrast CT scan in the transaxial plane. Arrow heads point to venous collaterals. Large arrow points to gastrostomy tube.

A system of collateral veins—shown in Figure 2—draining into the splenic vein and anterior abdominal wall veins was seen, and these were prominent around the stoma site and stomach, explaining the recurrence of bleeding from that site. 

The infant depended on TPN, invasive ventilation, was continuously on inotropic support, had limited central vascular access, and showed signs of neurological deterioration. Due to the vascular access problems and severe gastrointestinal bleeding, the child was discussed with the national intestinal failure unit, but was considered not to be a candidate for small bowel transplantation. Following another massive respiratory and circulatory deterioration, intensive care interventions were withdrawn and the child eventually died in multiorgan failure.

*Case 2*. The second case is a boy born with gastrointestinal malformations including gastroschisis and duodenal and colonic atresia. Surgical interventions included first a tapering duodenoplasty, hemicolectomy, ileostomy formation, and gastrostomy insertion, then later adhesiolysis prior to reconnection of his bowel with an ileocolonic anastamosis. Dense adhesions were noted during this laparotomy. There was difficulty establishing a stable enteral feeding regimen for this patient and clinically he displayed signs of gastrointestinal dysmotility. His bowels habits were persistently erratic and were most often very loose and frequent. These problems were managed medically. 

**Figure 3:**
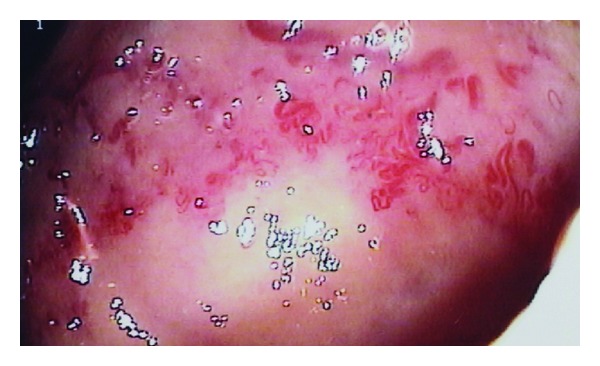
Torturous (corkscrew) vessels with dilatation at location of duodenoplasty.

At around 3 years of age the patient developed intermittent lower gastrointestinal bleeding. Routine coagulation blood tests including INR, prothrombin time, fibrinogen, APTT, and APTT ratio were normal. His full blood count was normal apart from intermittently low haemoglobin. After recurrent acute attendances to hospital with haematochezia requiring multiple blood transfusions an urgent endoscopy was arranged to identify the site of bleeding. Oesophagogastroduodenoscopy (OGD) revealed dilated blood vessels with angioectasia in the distal duodenum at the site of the previous duodenal surgery as shown in Figure 3.

**Figure 4:**
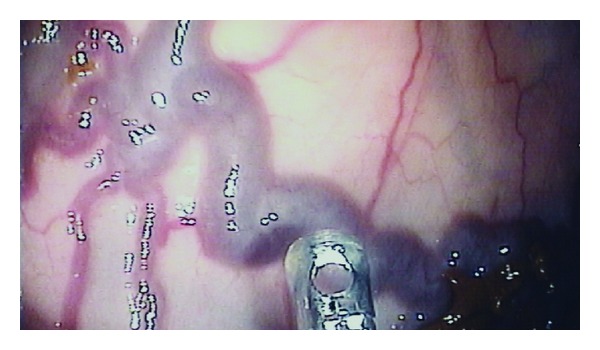
Large, torturous vessel at ileocolonic anastomosis. To demonstrate the size of the vessel. the tip of the forceps is used as a 2.8 mm reference size.

Colonoscopy to the transverse colon showed further venous dilatation and congestion with corkscrew angioectasia—shown in Figure 4—in the transverse colon and around the ileo-colonic anastamosis which was approximately 30 cm from the anal line. 

These areas of venous dilatation were assumed to be the source of bleeding although no active bleeding was ever seen. Meckel's diverticulum and arteriovenous malformations had also been considered as possible differential diagnoses, but were excluded by radioisotopic scan and angiography. The indication for video capsule endoscopy was discussed with experts from two other centres but the risk of capsule retention, even of a patency capsule, at the location of the duodenoplasty, with consecutive haemorrhage was considered too high. It was also considered inappropriate to apply laser or argon beam coagulation, as larger vascular convolutes were suspected in the mesentery and the intestinal wall.

**Figure 5:**
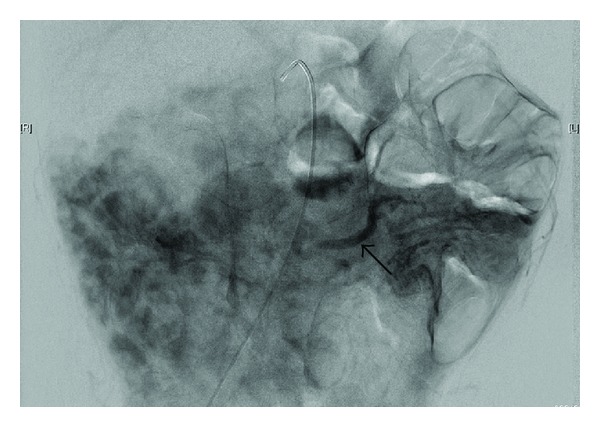
Angiogram showing prominent parenchymal contrast blush of the SMA vascular distribution, and prominent opacification of mesenteric veins (arrow).

Ultrasonography, angiography, and CT angiography were performed to further investigate the cause of this venous congestion and make assessments for potential treatment options. Doppler ultrasonography suggested these areas of dilatation involved the mesenteric veins. The precise origin of these vessels was not able to be determined during this ultrasound scan, however normal appearances of the portal and hepatic veins with normal blood flow was demonstrated. Angiography identified a filling defect over 5 cm of the SMV, and its patency could not be confirmed. Venous pooling was also seen as shown by the parenchymal “blush” seen in Figure 5. 

**Figure 6:**
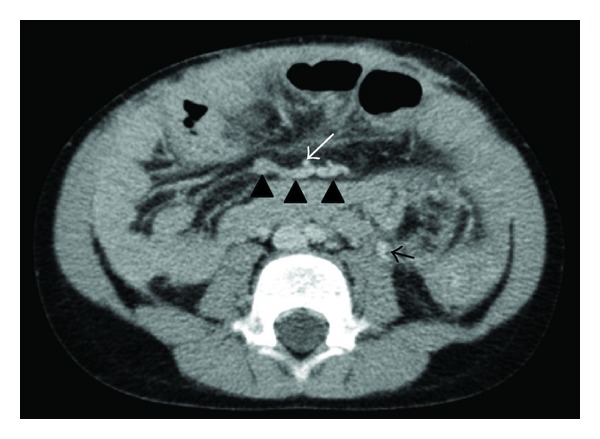
Post IV contrast transverse axial CT scan: SMA (white arrow), no normal SMV demonstrated, confluence of collateral veins (black arrow heads), large inferior mesenteric vein that drains to the splenic vein (black solid arrow).

The inferior mesenteric vein (IMV) was seen to be enlarged. An occlusion of the SMV was suggested. CT angiography confirmed these findings and demonstrated collateral veins draining into the IMV—these collateral vessels are shown in Figure 6. No normal SMV was seen. In addition to this an area of dense fibrous tissue, most likely the result of multiple abdominal surgeries, seen surrounding both the SMA and SMV gave the suggestion of extraluminal occlusion of the SMV as well as an intrinsic component. 

The patient had three further episodes of lower gastrointestinal bleeding at one month, two months, and 4 months after initial endoscopy and the identification of venous congestion. These were haemodynamically significant and required blood products. He also underwent further surgery for adhesiolysis for treatment of mechanical ileus. His liver function and clotting function tests remained within normal limits throughout this period. Notably, following these life-threatening problems the patient could be established on a good nutritional regime and was thriving with regular input from a multidisciplinary gastrointestinal team. He has no family history of inherited coagulopathy and investigations for inherited thrombophilia have not been undertaken. 

The patient was repeatedly assessed for surgical options at the national intestinal failure unit to bypass the SMV thrombosis and release the pressure on the intestinal varices. A Mesorex-shunt was not indicated due to the anatomical location of the thrombosis. The two surgical options, mesenteric-renal shunt or small bowel transplantation, were considered to carry a very high mortality risk for a child with established enteral feeding and reasonably good quality of life. Three years later on regular followup, there have been no further episodes of gastrointestinal bleeding, the child is thriving, attending school and is on routine outpatient surveillance. Doppler ultrasound investigations show good portal flow, no signs of hypersplenism, and the child has no anaemia.

## 3. Discussion

We believe that our case reports are the first paediatric cases to describe SMV thrombosis as the cause of intestinal varices leading to severe gastro-intestinal bleeding at a surgical site. Both the cases summarised above had undergone complex surgical interventions, and developed SMV thrombosis following these interventions. The cardinal features illustrated here are not listed among common causes of gastrointestinal bleeding in children, according to the current literature [4, 5]. However, in adults, SMV thrombosis and other intra-abdominal venous thromboses have been described in patients who have undergone abdominal surgery. It is well described postsplenectomy, when thrombocytosis can occur adding to the post-operative risk of thrombosis [6]. Isolated case reports in paediatrics have described mesenteric venous thrombosis following surgery, such as post colectomy [7]. Other cases of SMV thrombosis have been reported in children with procoagulation risk factors such as inherited thrombophilias and oral contraceptive use [8, 9]. In all these cases the patients presented acutely and were found to have ischaemic bowel requiring resection. In contrast, the two patients we describe above had a more insidious presentation, with episodes of bleeding over a longer period of time, and neither had evidence of acute bowel ischaemia on endoscopic examination. Neither of our two patients had an underlying thrombophilia diagnosed. 

Interestingly, in our cases, venous congestion and, most notably varices, occurred around the sites of previous surgery, at the gastrostomy site in the first case, and at the site of intestinal anastamosis in the second. This has not been explicitly described previously in case reports. The development of extensive venous collaterals avoided acute intestinal ischaemia and the need for bowel resection. This was fortunate particularly in the second case where extensive bowel resection had already been necessary. The investigation of these patients involved first ultrasonography and then CT angiography. In both patients normal blood flow in the portal and hepatic veins was seen on ultrasonography, but full assessment of the mesenteric veins could not be made without CT angiography. This highlights the importance of fully visualising the mesenteric vessels in the assessment of such cases. 

Treatment of SMV thrombosis as described in isolated case reports usually involves anticoagulant therapy to directly treat the thrombosis and for long term management of an underlying prothrombotic state. The presence of venous congestion and varices in our cases made the risk of bleeding significant and neither received anticoagulants. Alternative therapeutic options were not explored in the first case due to the patient's comorbidities and prognosis but were considered in the second. Treatment strategies with interventional radiology have been described, predominantly in adult patients, with success. A case series of 46 patients with portal or SMV thrombosis, all of whom had acquired risk factors for thrombosis describes excellent results following direct thrombolysis through both transjugular intrahepatic portosystemic shunts (TIPS) and percutaneous transhepatic portal vein cannulation [10]. This was only performed in patients with no demonstrable lateral branch angiogenesis on CT angiography and these patients were identified 1-2 weeks after the onset of symptoms. Patients with evidence of angiogenesis were managed with indirect thrombolysis with access via the femoral or radial artery. This was a largely successful intervention, but less so than direct thrombolysis. 

SMV thrombosis continues to be a rare clinical entity, but developments in clinical imaging allow its diagnosis. Early recognition of symptoms and early diagnosis is important as chronicity and the development of collateral blood vessels increases bleeding risk and limits management options. The continuing development of minimally invasive interventional radiology makes SMV thrombosis a condition which potentially could be successfully treated without the complications associated with surgical management. Identifying the underlying pathology which leads to thrombosis remains crucial for the long-term management and well-being of these patients.
